# RT-QuIC-based detection of alpha-synuclein seeding activity in brains of dementia with Lewy Body patients and of a transgenic mouse model of synucleinopathy

**DOI:** 10.1080/19336896.2020.1724608

**Published:** 2020-02-10

**Authors:** Jung-Youn Han, Hyung-Sup Jang, Alison J. E. Green, Young Pyo Choi

**Affiliations:** a Laboratory Animal Center, Division of Research Strategy, Korea Brain Research Institute, Daegu, Republic of Korea; b National CJD Research & Surveillance Unit, Centre for Clinical Brain Sciences, School of Clinical Sciences, University of Edinburgh, Edinburgh, UK

**Keywords:** RT-QuIC, a-synuclein, seeded aggregation

## Abstract

RT-QuIC is a shaking-based cyclic amplification technique originally developed in the prion field to detect minute amounts of scrapie prion protein (PrP^Sc^). In this study, we applied the RT-QuIC assay to investigate a-synuclein (a-syn) seeding activity in brains of Dementia with Lewy Body (DLB) patients and in brains of G2-3 transgenic mice expressing human a-syn with A53T mutation. The results show that a-syn seeding activity varies between patients with detectable dilutions ranging from 10^−3^ to 10^−8^ dilutions of brain tissue and is stable under exposures to the cycles of freezing, thawing and sonication. A53T a-syn aggregates from G2-3 transgenic mice greatly favoured A53T recombinant human a-syn as substrates in comparison to wild-type a-syn, suggesting that conformations for wild-type a-syn to be able to adopt are not compatible with that of A53T aggregates from G2-3.

## Introduction

Alpha-synuclein (a-syn) is a well-conserved, soluble and natively unfolded protein consisting of 140 amino acids that is highly expressed in the nervous tissue [1]. a-syn is predominantly localized to the presynaptic nerve terminals and thus thought to be play an important role in synaptic vesicle biology [2,3]. a-synucleinopathies, which include Parkinson’s disease (PD), dementia with Lewy bodies (DLB) and multiple system atrophy (MSA), are characterized by the accumulation of phosphorylated, insoluble a-syn aggregates in neuronal or glial cells [3,4]. While these a-syn deposits are found as glial cytoplasmic inclusions in MSA [5,6], they are present in neuronal cell bodies and in dystrophic neurites of selected population of neurons (known as Lewy bodies and Lewy neuritis, respectively) in PD and DLB [7]. Several missense mutations in the *SNCA* gene that encodes for a-syn, and duplications or triplications of the wild-type *SNCA* gene were all found to be associated with familial PD and/or DLB [8–12].

It has been shown that fibrillar a-syn aggregates can replicate by conferring their disease-associated conformation to native a-syn [13]. The replication and spread of a-syn aggregates along neuroanatomically connected regions results in a stereotypical progression of Lewy pathology in diseased human brains [14]. This prion-like propagation of a-syn fibrils has been extensively investigated by inducing a-synucleinopathies in animal models [15,16] as well as in cultured cells [17,18]. Notably, as in prions, the presence of distinct a-syn strains has been demonstrated in transmission studies of a-synucleinopathies into cultured cells and/or transgenic mice [19,20].

RT-QuIC (real-time quaking-induced conversion) is a highly sensitive biochemical assay originally developed in the prion field to detect minute amounts of scrapie prion protein (PrP^Sc^) in brain tissue or body fluids [21,22]. In the RT-QuIC assay, a mixture of test specimen and recombinant prion protein in a multiwell plate is intermittently shaken to amplify prion protein aggregates in a seed-dependent manner [23]. Real-time monitoring of fluorescence signal of thioflavin T (ThT) that binds to β-sheet-rich structures in amyloid fibrils allows the detection of prion seeding activity in tested samples [24]. In recent years, successful adaptation of the RT-QuIC assay for a-synucleinopathies has been reported by several groups [25–28]. Similar to the prion protein assay, a-syn RT-QuIC was able to detect a minute amount of disease-associated a-syn in tissue lysates or in body fluids from patients with a-synucleinopathies [29,30]. In this study, we investigated a-syn seeding activity in brains of DLB patients or in brains of transgenic mice expressing human A53T a-syn [31] using RT-QuIC assay adapted for a-syn [26,27]. Our results show that a-syn seeding activity is often variable between DLB cases, and A53T a-syn aggregates from transgenic mice greatly favours recombinant A53T a-syn in comparison to wild-type a-syn (i.e. lacking A53T mutation) as substrate in the seeded aggregation assay.

## Materials and methods

Human brain tissue was obtained from the Edinburgh Brain and Tissue Bank (EBTB) in Edinburgh, Scotland, UK. Brain samples from seven patients affected by DLB and from six controls (Con) were used in this study (Table 1). Frozen brain samples pseudoanonymized using the Bank’s reference number system were sent to Korea Brain Research Institute (KBRI) without any patient identifiable data. Ethical approvals for the acquisition and use of human brain samples were obtained from KBRI’s Institutional Review Board. Brain tissue was homogenized in nine volumes of phosphate-buffered saline (PBS, pH 7.4) containing complete EDTA-free protease inhibitors and phosphatase inhibitors (Roche Applied Science), by using the Precellys 24 tissue homogenizer (Bertin instrument). The 10% (w/v) homogenates were clarified by centrifugation at 2,000 × g for 2 min and the supernatants were transferred to new tubes and kept in aliquots at −80°C until investigated. Unless specified otherwise, the aliquots were used only once after thawing and any remaining ones were discarded.

**Table 1.** Summary of the α-syn RT-QuIC assay for multiple human brain tissue.

Case ID	Gender	Age at death^1)^	Neuropathological diagnosis	Brain region^2)^	Detectable dilution
Con1	F	79	Cerebrovascular disease	Frontal cortex	10^−3^
Con2	M	76	Normal	Frontal cortex	ND^3)^
Con3	M	29	Normal	Frontal cortex	ND
Con4	M	39	Normal	BA9^4)^	ND
				BA39	ND
Con5	F	74	Normal	BA9	ND
				BA39	ND
Con6^5)^	M	83	–	Frontal cortex	ND
				Thalamus	ND
DLB1	M	78	DLB	Frontal cortex	10^−8^
				Thalamus	10^−5^
DLB2	M	55	DLB	Frontal region	10^−7^
				Parietal region	10^−6^
DLB3	M	55	DLB	Frontal cortex	10^−3^
				Thalamus	10^−5^
DLB4	F	86	DLB	BA9	10^−7^
				BA39	10^−6^
DLB5	M	60	DLB	BA9	10^−8^
				BA39	10^−7^
DLB6	M	65	DLB	BA9	10^−8^
				BA39	10^−7^
DLB7	M	61	DLB	BA9	10^−7^
				Parietal region	10^−7^

1) Age of death in years

2) The information on brain regions were described as provided by the EBTB.

3) ND = not detectable at 10^−3^ dilution

4) BA = Brodmann area

5) This case was initially sent to us as one of DLB samples. Despite multiple RT-QuIC trials, however, a-syn seeding activity was not detectable in this case (even at 10^−2^ dilution). Given that EBTB’s re-examination of the brain tissue from this case revealed no convincing a-syn pathology, we categorized this case as control in this study.

The transgenic mouse line G2-3 expressing human a-syn with A53T mutation under the control of murine prion protein promoter was described previously [31,32]. The use of G2-3 mice was approved by KBRI’s Institutional Animal Care and Use Committee. Mice transgenic for human A53T a-syn and non-carrier controls were euthanized at 10–11 months, and their brains were recovered. Caudal parts (brainstem, pons and midbrain) of these brains were separated and homogenized as described above. Clarified brain homogenates were stored in aliquots at −80°C until use.

RT-QuIC was performed as described previously [26] with modifications [27]. Briefly, seeded a-syn aggregation was conducted in 100 mM phosphate buffer (pH 8.2) containing 10 μM recombinant full-length (140 amino acids) human α-syn protein (rPeptide) and 10 μM thioflavin T (ThT) in 96-well black plates with clear bottom (Nalgene Nunc). Recombinant human α-syn protein (recSyn), either wild-type or A53T mutant protein, was supplied in the form of lyophilized powder and stored in freezer until use. On the day of experiment, the recSyn was resuspended in water at a concentration of 100 μM, filtered through a 50 kDa filter (Millipore) for 10 min at 15,000 × g, and added to a reaction mixture at a final concentration of 10 μM. Any remaining resuspended recombinant a-syn solution was discarded. With regard to seeds, aliquots of brain homogenates stored at −80°C were thawed and extensively sonicated with 5 cycles of 30 s sonication and 30 s rest in Microsonix 4,000 (Sonix) set at 80% potency. Subsequently, sonicated brain homogenates were serially diluted and used as seeds at various dilutions. Dilutions were expressed in relation to brain; for example, 10^−3^ dilution is equivalent to 0.1% brain homogenate. Remaining brain homogenates after use as seeds were discarded. In some experiments to investigate the stability of a-syn seeding activity, sonicated brain homogenates were re-frozen at −80°C, thawed after day(s) and sonicated again; this set of freezing, thawing and sonication was repeated up to four times. The brain homogenates exposed to the freezing/thawing/sonication cycle from one time to four times were serially diluted and used as seeds. Each well of the plate preloaded with 4 glass beads (diameter: 1.0 ~ 1.25 mm) was given 100 μL of reaction mixture seeded with 2 μL of brain homogenates. Reactions for individual samples were prepared in triplicate. The plate was then incubated at 37°C with cycles of 1 min shaking (400 rpm, double orbital) and 1 min rest in a FLUOstar Omega plate reader (BMG Labtech). The plate reader measures ThT fluorescence in relative fluorescence units (rfu) and is saturated at 260,000 rfu. ThT fluorescence was measured at 1-h intervals from bottom of wells (440 nm excitation and 480 nm emission, gain of 2,000, 20 flashes per well). Fluorescence values at each reading point were shown as average for triplicate wells on the graph. A ThT fluorescence threshold was determined as described previously [27,33] with minor modifications, by taking the average fluorescence of the first five readings for all samples plus five standard deviations. When wild-type recSyn and A53T recSyn were used as substrate within the same plate, average fluorescence was obtained separately based on the type of recombinant protein. Samples were considered positive when at least two of three replicates crossed the threshold fluorescence value at 60^th^ reading (approximately 60.5-h incubation).

## Results

**Figure 1.**
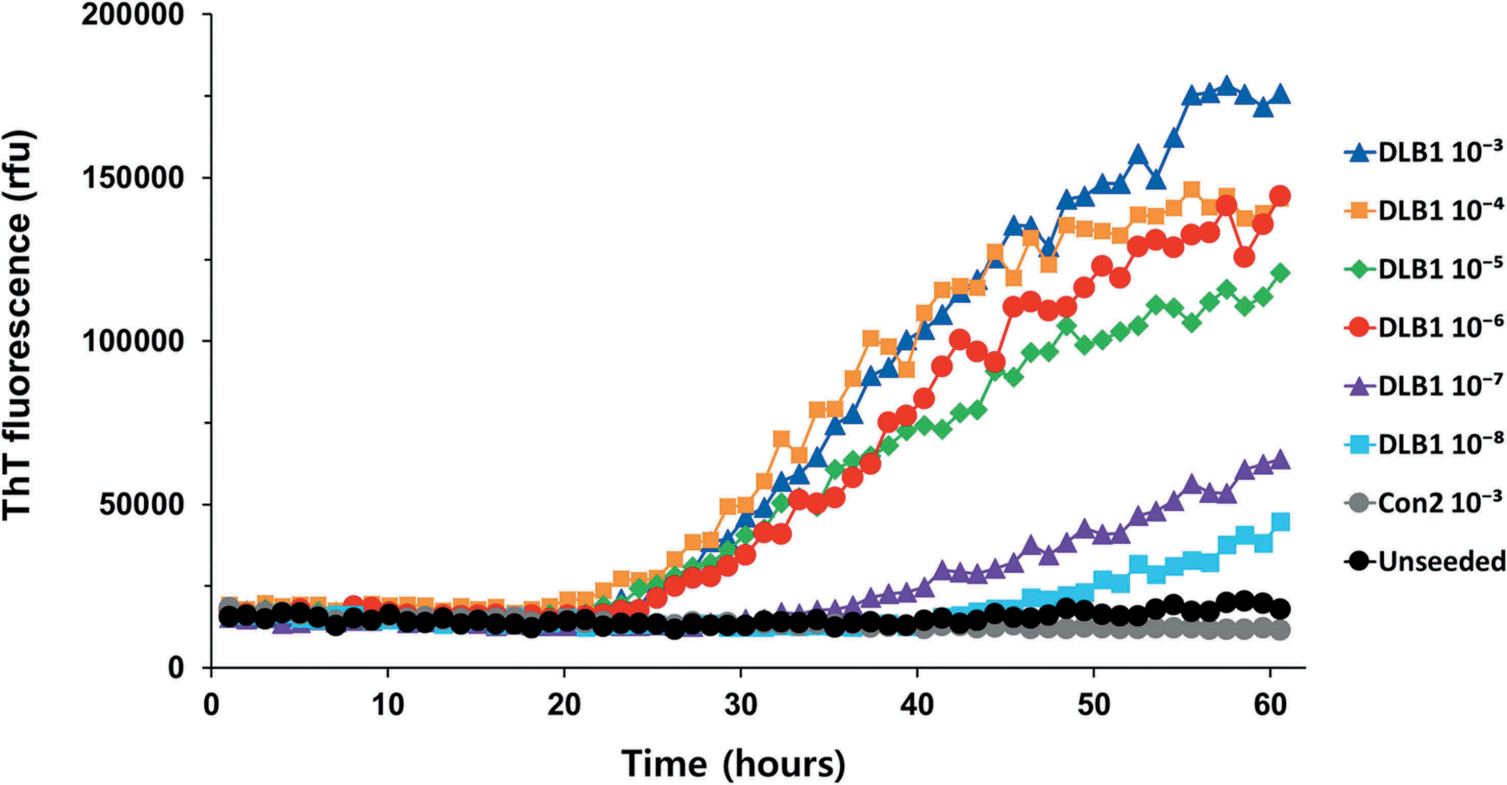
RT-QuIC detection of a-syn seeding activity in DLB using wild-type human a-syn as substrate. Serial dilutions (10^−3^ to 10^−8^) of human frontal cortex brain tissue derived from a DLB patient (DLB1) were used to seed RT-QuIC reactions with wild-type recSyn as substrate. Reactions seeded with 10^−3^ dilution of frontal cortex tissue from a non-demented patient (Con2) were included as controls. Unseeded reactions are also shown. ThT fluorescence was measured every 1 h and average values from triplicate wells were plotted as a function of time.

The optimal conditions of RT-QuIC assay were initially investigated using wild-type recSyn as substrate and frontal cortex (FC) tissue of DLB1 or Con2 brains as seeds. While the ThT fluorescence signals from unseeded reactions or reactions seeded with 10^−3^ dilution of Con2 FC tissue remained at baseline values throughout the assay, reactions seeded with 10^−3^ to 10^−6^ dilutions of DLB1 FC tissue gave responses rising above baseline after a lag phase of approximately 20 h (Figure 1). Positive responses for α-syn seeding activity were seen in reactions seeded with up to 10^−8^ dilution of DLB1, although there were much longer lag phases and lower maximal responses in reactions seeded with higher dilutions (10^−7^ to 10^−8^).

We then investigated RT-QuIC seeding activity in more cases of DLB and non-demented controls using wild-type recSyn as substrate. Positive RT-QuIC responses were seen in all DLB cases. Maximal dilution allowing us to detect α-syn seeding activity varied between regions within the same brain as well as between DLB cases, ranging from 10^−3^ to 10^−8^ dilutions of the brain tissue (Table 1). Reactions seeded with brain homogenates prepared from control cases gave flat responses in the assay, except for one case (Con1) in which seeding activity was detectable at a 10^−3^ dilution of the brain tissue (Table 1).

**Figure 2.**
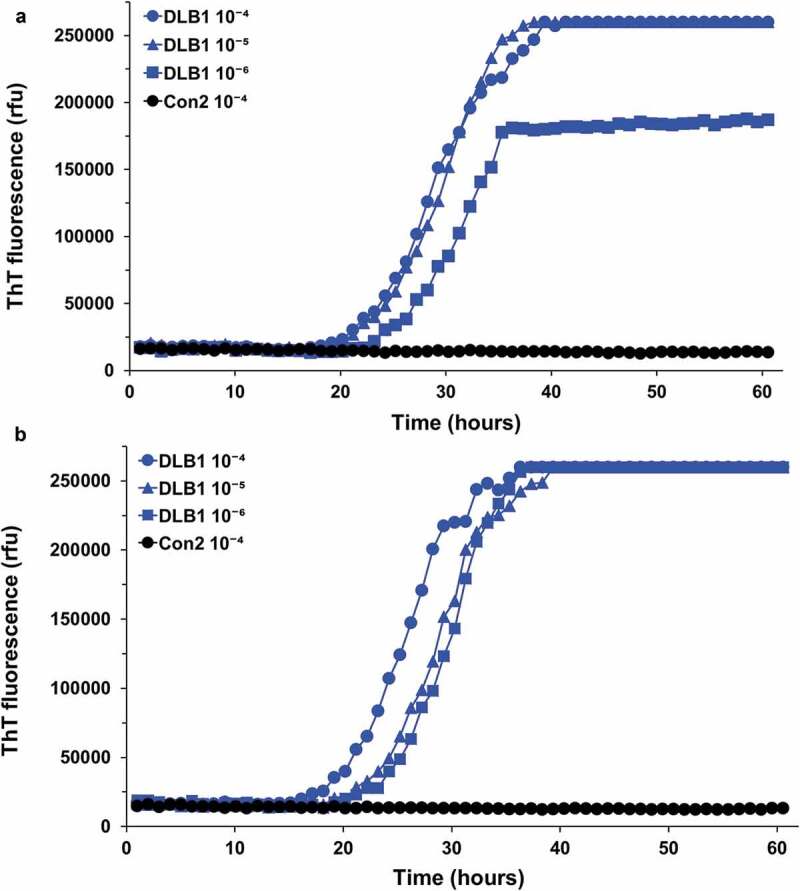
a-syn seeding activity detected by the RT-QuIC assay is stable after repeated freezing/thawing/sonication cycles. Aliquots of frontal cortex brain homogenates, prepared either from DLB1 or Con2 patient, were repeatedly subjected to the cycle of freezing (−80°C), thawing and sonication. The designated dilutions of the DLB1 (10^−4^ to 10^−6^) or Con2 (10^−4^) frontal cortex brain tissue exposed to the cycle one time (a) or four times (b) were used to seed RT-QuIC reactions with wild-type recSyn as substrate. ThT fluorescence was measured every 1 h and average values from triplicate wells were plotted as a function of time.

Next, in order to examine the stability of a-syn seeding activity under laboratory conditions, FC brain homogenates from DLB1 or Con2 patient were exposed to the cycle of freezing, thawing and sonication up to four times with one-day or longer intervals between cycles. Subsequently, the DLB1 or Con2 brain homogenates were serially diluted and used as seeds in the RT-QuIC assay. The kinetics of RT-QuIC reactions seeded with 10^−4^ to 10^−6^ dilutions of the DLB1 FC tissue was overall similar regardless of the number of freeze/thaw cycles (Figure 2 and data not shown). It took approximately 20 h to produce responses rising above baseline values, and generally 30 to 40 h to reach maximal rfu signals. These results indicate that α-syn seeding activity in DLB brain tissue was stable and resistant to several cycles of freezing, thawing and laboratory-conditioned sonication.

**Figure 3.**
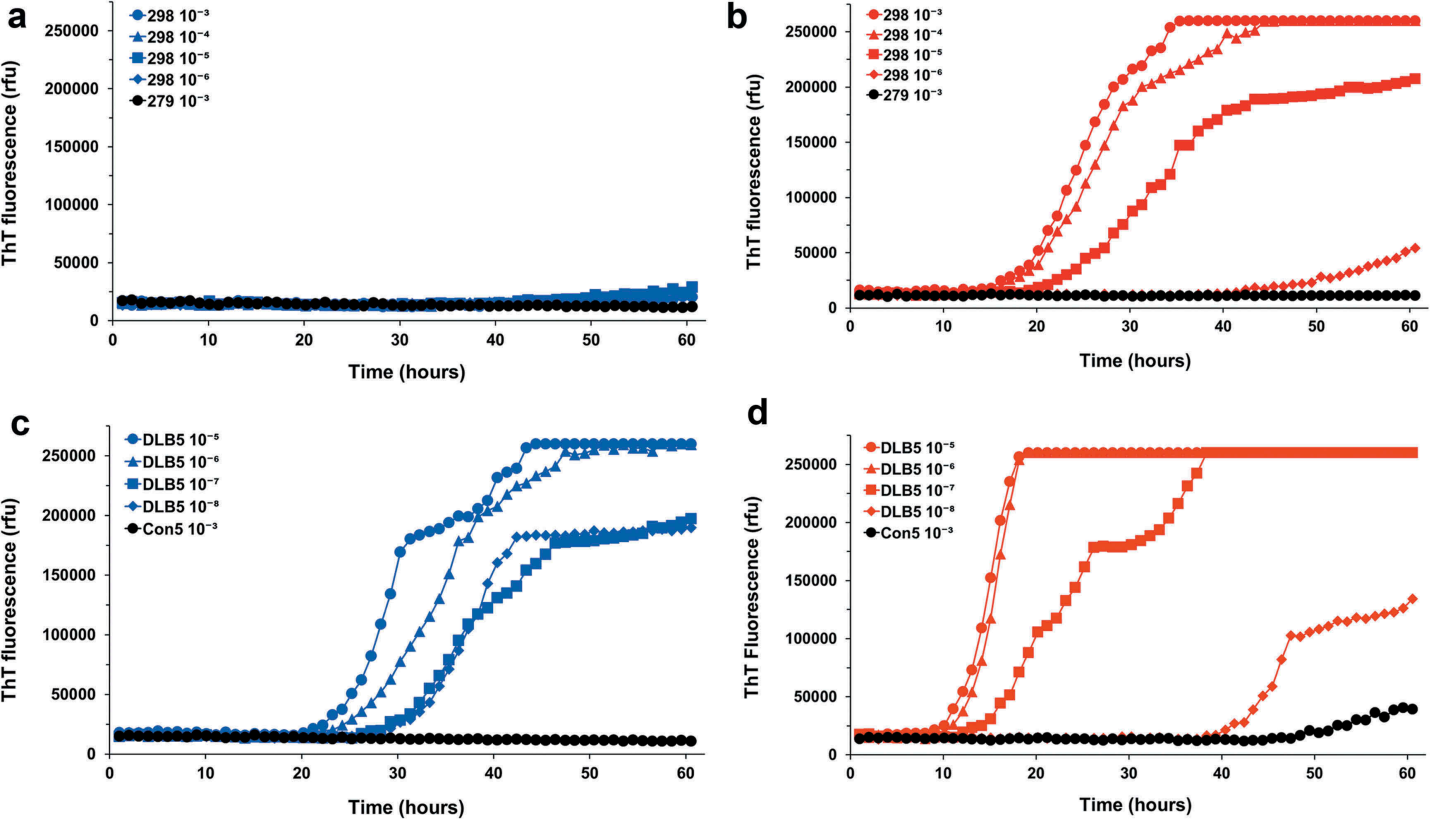
A53T a-syn seeding activity in the G2-3 transgenic mouse is much more efficiently detected using recombinant A53T recSyn than wild-type recSyn. The designated dilutions of 298 G2-3 brain tissue (a and b, 10^−3^ to 10^−6^) or the DLB5 BA9 brain tissue (c and d, 10^−5^ to 10^−8^) were used to seed RT-QuIC reactions using wild-type (a and c) or A53T (b and d) recSyn as substrate. As controls, reactions were also seeded with a 10^−3^ dilution of Con5 BA9 brain tissue or 279 non-carrier mouse brain tissue. ThT fluorescence was measured every 1 h and average values from triplicate wells were plotted as a function of time.

We then investigated A53T a-syn seeding activity in RT-QuIC using brain homogenates derived from G2-3 transgenic mice expressing human α-syn with A53T mutation. When wild-type recSyn lacking A53T mutation was used as substrate for seeded conversion in the assay, positive responses were usually seen at 10^−3^ and/or 10^−4^ dilutions of G2-3 mouse brains. Unlike those seeded with DLB brain homogenates, however, reactions seeded with G2-3 mouse brain homogenates gave positive responses that were distinctively low and inconsistent between experiments and/or replicate wells with mostly only two of three wells rising above background levels (Figure 3(a) and data not shown).

Since there was a mismatch at amino acid residue 53 between substrate and seed, we wondered whether this mismatch might be associated with low and inconsistent responses in the assay seeded with G2-3 brain homogenates. To investigate this possibility, we conducted a homologous RT-QuIC assay by replacing wild-type recSyn with A53T recSyn. As shown in Figure 3(b), a drastically different RT-QuIC response was seen when A53T recSyn was used as substrate. In reactions seeded with 10^−3^ and 10^−4^ dilutions of G2-3 brain tissue, the increase of ThT fluorescent signal rising above baseline values appeared as early as 15 h and reached a plateau in 30–40 h. The reactions seeded with higher dilutions of G2-3 seed (10^−5^ and 10^−6^) were slower and did not reach the plateau throughout the 60-h reaction time, but their positive responses were evident (Figure 3(b)). In the reactions seeded with DLB brain homogenates, the kinetics of conversion was basically similar between WT recSyn and A53T recSyn, except for shorter lag times with A53T recSyn (Figure 3(c,d)). It was also noticeable, however, that spontaneous positive response was occasionally seen in the reactions using A53T recSyn as substrate (Figure 3(d) and data not shown).

## Discussion

RT-QuIC, which is a shaking-based cyclic amplification technique originally developed in the prion field [21,22,34], has been adapted for a-synucleinopathies in recent years by several groups [26,27,30,33,35]. Using a-syn RT-QuIC, we examined a-syn seeding activity in the brains of DLB patients. As shown in Table 1, a-syn seeding activity was found to be often variable between DLB brains with detectable dilutions ranging from 10^−3^ to 10^−8^ dilutions of brain tissue. This a-syn seeding activity was stable against repeated exposures to the cycle of freezing, thawing and sonication, which is consistent with stability and resistance of a-syn seeds to various inactivation methods [36–38]. Nonetheless, it remains to be explored whether a-syn seeding activity would be stable at higher dilutions of DLB brains (for example those at 10^−7^ or 10^−8^).

We detected a-syn seeding activity in one non-demented control brain (Con1). This brain was sent to us as one of the non-demented controls from the EBTB, but was found on the information sheet to have cerebrovascular disease including lacunar infarct and small vessel lipohyalinosis. Although the deposition of aggregated a-syn was not recorded on this information sheet, a-syn seeding activity in the Con1 brain may be associated with high prevalence of a-syn pathology in the brains of cognitively normal elderly [39]. Given that the number and region of controlhuman brains examined in this study were relatively small and limited, investigation of more cases of non-demented human controls could be useful in identifying the prevalence of pathogenic a-syn seeds in cognitively normal elderly.

RT-QuIC responses in reactions seeded with brain homogenates from G2-3 transgenic mice expressing A53T mutant a-syn differed markedly between two recSyn sequences, wild-type and A53T. While homologous reactions (A53T seeds and A53T substrates) lead to seeded aggregation with high efficiency, heterologous reactions (A53T seeds and wild-type substrates) did not support the conversion efficiently. In contrast, wild-type a-syn aggregates supported the seeded conversion of both A53T and wild-type recSyn in RT-QuIC. *In vitro* aggregation of wild-type or A53T recSyn was known to form morphologically and biochemically distinct fibrils [40] and these structural properties were reported to be transmitted in seeding experiments in a seed-dependent manner [41,42]. In a similar context, whether conformation favoured by soluble monomeric recSyn is compatible with that of seeds was suggested to be a critical factor in inducing seeded aggregation [43]. Thus, the poor ability of A53T aggregates in inducing seeded aggregation of wild-type recSyn is probably associated with conformational limitation for wild-type recSyn to be able to adopt in the given condition. In comparison, given the results in this study, the conformation of wild-type a-syn aggregates is thought to be compatible with conformations for both wild-type and A53T recSyn to be able to adopt in the experimental condition.
